# Adapting open-source drone autopilots for real-time iceberg observations

**DOI:** 10.1016/j.mex.2018.09.003

**Published:** 2018-09-06

**Authors:** Daniel F. Carlson, Søren Rysgaard

**Affiliations:** a Arctic Research Centre, Department of Bioscience, Aarhus University, 8000 Aarhus C, Denmark; b Department of Earth, Ocean, and Atmospheric Science, Florida State University, Tallahassee, FL 32306, USA; c Centre for Earth Observation Science, University of Manitoba, Winnipeg, MB R3T 2N2, Canada

**Keywords:** Low-cost, Real-time iceberg drift and orientation observations, Iceberg tracking, Iceberg drift and orientation, Operational iceberg management, Drone autopilot, Iceberg-ocean interaction

## Abstract

Drone autopilots are naturally suited for real-time iceberg tracking as they measure position and orientation (pitch, roll, and heading) and they transmit these data to a ground station. We powered an ArduPilot Mega (APM) 2.6 with a 5V 11 Ah lithium ion battery (a smartphone power bank), placed the APM and battery in a waterproof sportsman’s box, and tossed the box and its contents by hand onto an 80 m-long iceberg from an 8 m boat. The data stream could be viewed on a laptop, which greatly enhanced safety while collecting conductivity/temperature/depth (CTD) profiles from the small boat in the iceberg’s vicinity. The 10 s position data allowed us to compute the distance of each CTD profile to the iceberg, which is necessary to determine if a given CTD profile was collected within the iceberg’s meltwater plume. The APM position data greatly reduced position uncertainty when compared to 5 min position data obtained from a Spot Trace unit. The APM functioned for over 10 h without depleting the battery. We describe the specific hardware used and the software settings necessary to use the APM as a real-time iceberg tracker. Furthermore, the methods described here apply to all Ardupilot-compatible autopilots. Given the low cost ($90) and ease of use, drone autopilots like the APM should be included as another tool for studying iceberg motion and for enhancing safety of marine operations.

• Commercial off-the-shelf iceberg trackers are typically configured to record positions over relatively long intervals (months to years) and are not well-suited for short-term (hours to few days), high-frequency monitoring
• Drone autopilots are cheap and provide high-frequency (>1 Hz) and real-time information about iceberg drift and orientation
• Drone autopilots and ground control software can be easily adapted to studies of iceberg-ocean interactions and operational iceberg management

**Specifications Table**

**Table:** 

Subject area	• *Earth and Planetary Sciences* • *Energy* • *Engineering* • *Environmental Science*
More specific subject area	*Cryosphere, Oceanography, Ice Hazards, Oil and Gas*
Method name	*Low-cost, real-time iceberg drift and orientation observations*
Name and reference of original method	*Iceberg tracking was first pioneered by the International Ice Patrol -*https://cgaviationhistory.org/1946-international-ice-patrol/*and also summarized in – Icebergs: Their Science and Links to Global Change by G.R. Bigg* [7]*. Cambridge University Press*
Resource availability	*All required documentation to use an open source autopilot as an iceberg tracker can be found here:*http://ardupilot.org/

## Method details

### Background

Icebergs play an important role in Polar ocean environments [[1], [2], [3]], and can also threaten marine infrastructure [[4], [5], [6]]. Observations of iceberg translation and rotation are relatively rare, as are oceanographic observations in the vicinity of drifting icebergs. These observations are necessary, however, to improve our understanding of iceberg-ocean interactions and to develop reliable models of iceberg drift and deterioration. For a review of iceberg science we refer the reader to Bigg [7].

**Fig. 1:**
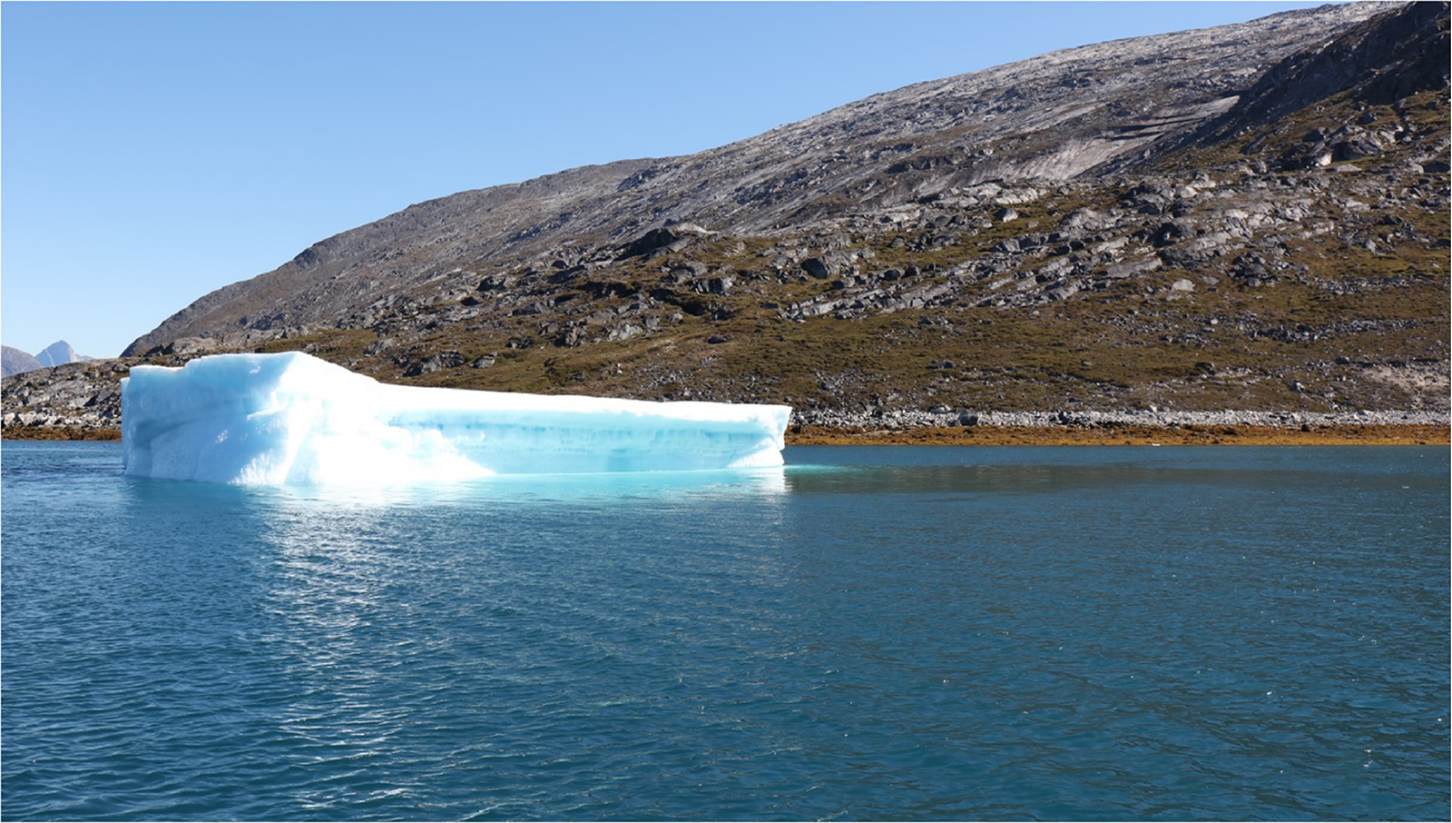
The melt water plume from this grounded iceberg is evident on the surface of the ocean as a calm, glassy region. It is most visible to the right of the iceberg.

Oceanographic observations around icebergs typically attempt to capture the signature of the iceberg’s melt water plume in water temperature and salinity [2,8,9]. The structure of an iceberg’s melt water plume depends on the relative velocity between the iceberg and ocean currents [10] and in stratified waters the plume behavior also depends on the density difference between the plume and the saltier water below [8,9]. Attached (detached) iceberg plumes generally occur when the relative velocity is small (large; [9]) and strong vertical velocity shear can result in more complicated plume behavior [10]. In some cases, particularly during very calm conditions, the surface signature of the melt water plume may be clearly visible (Fig. 1). During windy and wavy conditions, however, the location of the meltwater plume may not be immediately apparent to the eye. Similarly, real-time information about oceanographic conditions (e.g., stratification and velocity shear) and iceberg drift may not be available to researchers, which makes field assessments of plume structure difficult, if not impossible. Instead, plume behavior is usually assessed from field or laboratory data. Observations of the direction and speed of the iceberg’s drift can aid, therefore, in the interpretation of oceanographic measurements.

Oceanographic observations of iceberg meltwater plumes consist of conductivity/temperature/depth (CTD) profiles, at a minimum, and can include many other variables, including water velocity profiles, fluorescence, photosynthetically available radiation (PAR), dissolved oxygen (DO), turbidity, and nutrients, for example. The micro-scale mixing due to the buoyant meltwater plume and the displacement of water by the iceberg keel may also be inferred using a turbulence profiler. Ocean profile measurements are typically geolocated using data from a global positioning system (GPS) or a global navigation satellite system (GNSS) receiver and the date and time are most often recorded in coordinated universal time (UTC).

Interpretation of oceanographic measurements is complicated by the fact that the position of the iceberg varies during the interval over which the oceanographic measurements are conducted [8]. Therefore, the iceberg trajectory should be recorded with sufficient temporal resolution and spatial accuracy to minimize the uncertainty in the estimates of relative separation between the oceanographic measurement and the iceberg’s position at a given time. Ideally, the trajectory should be recorded in the same spatial and temporal reference frames as the oceanographic measurements.

Modern iceberg tracking methods include radar [[11], [12], [13], [14], [15]] and GPS beacons [[16], [17], [18], [19], [20], [21]]. Large icebergs (>1 km) can be tracked with satellites (e.g., [22]) but given the relatively low temporal resolution of satellite measurements we do not consider their use here. Radar-derived trajectories are typically used for operational iceberg management [13,14,23] and obviously requires the vessel or offshore platform to be within detection distance of the iceberg long enough to construct a trajectory. GPS beacons are typically used for studies of iceberg trajectories over relatively long periods of 30 days to over 1 year and transmit their data at intervals ranging from 5 min (e.g., [16]) to 1 h (e.g., [21]). Operational ice management may utilize radar and GPS beacons, as well as other sources of data, depending on the distance, and travel time, of the ice hazards to the infrastructure in need of protection [6].

The measurement interval of GPS beacons is usually selected as a compromise between the temporal resolution of the position measurements and the overall tracker lifetime. Measurement intervals of 1 h are acceptable for studies of iceberg drift pathways and flow in the upper water column in fjords over sufficiently long periods of time. Higher frequency iceberg position measurements, however, are needed to interpret oceanographic measurements that attempt to observe iceberg-ocean interactions.

The ship’s radar could be used to obtain the distance of oceanographic measurements to the iceberg. Conventional radars available on most ships usually do not log data, requiring either the use of custom software packages [13,24] or an operator to manually record data [12,14]. Therefore, radar tracking solutions can be expensive or labor-intensive and also rely on a functioning radar. Oceanographic measurements could be conducted in regions that are covered by land-based radars [11,15] but such systems are rare in Polar regions.

### Motivation

The authors were motivated to solve the problem of reducing the uncertainty in distance estimates between a drifting iceberg and the locations of oceanographic measurements because such uncertainties arose when analyzing data that were collected near Nuuk, Greenland in August 2016. In this case, an expendable ice tracker (EXITE; [16]) was used to observe an iceberg’s trajectory. The EXITE beacon records GPS position at 5 min intervals using a Spot Trace, which transmits data via satellite using the GlobalStar constellation (see [16] for details). The GPS and GlobalStar antennae on the EXITE require a clear view to the sky and gaps of 15 min up to several days were common [16] and were most likely due to changes in orientation of either the EXITE or its host iceberg. EXITE data are visible in near-real-time through the Spot website, which requires an internet connection. As a result, field verification of proper EXITE functionality is usually not possible.

**Fig. 2:**
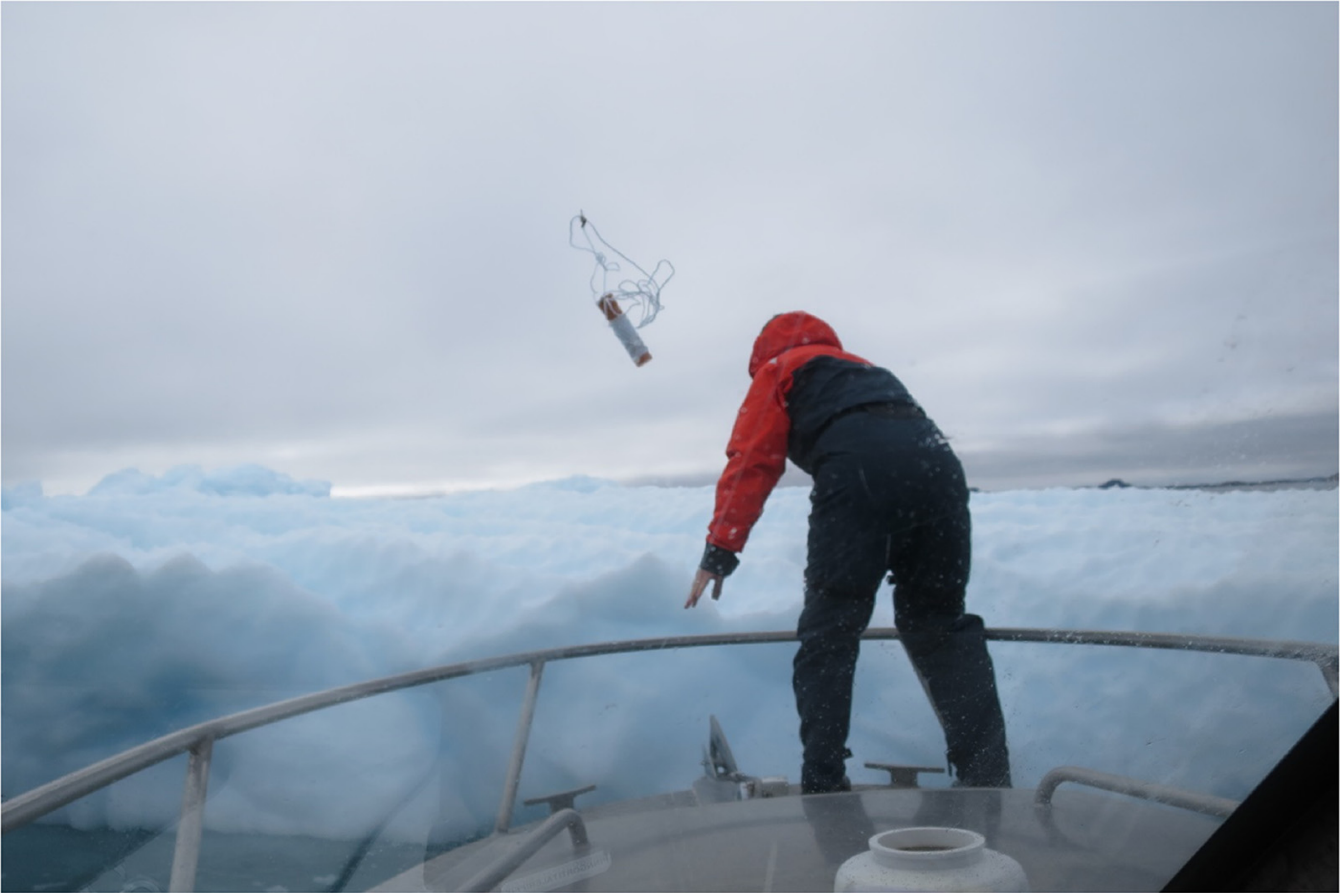
An expendable ice tracker (EXITE) deployed by hand. The EXITE was simply thrown from a small boat onto a drifting iceberg.

An EXITE beacon was deployed on a small iceberg (L ∼ 30 m) near the mouth of Godthabsfjord. The deployment simply consisted of throwing the EXITE beacon by hand from a small (8 m) vessel onto the iceberg (Fig. 2). This maneuver, while simple, requires approaching an iceberg and should only be attempted by experts. After the EXITE was deployed, CTD casts were collected around the drifting iceberg. A 50 min gap in the EXITE record occurred when most of the CTD measurements were collected. Even when the EXITE functioned properly, the 5 min measurement interval introduced large uncertainties in the distance.

**Fig. 3:**
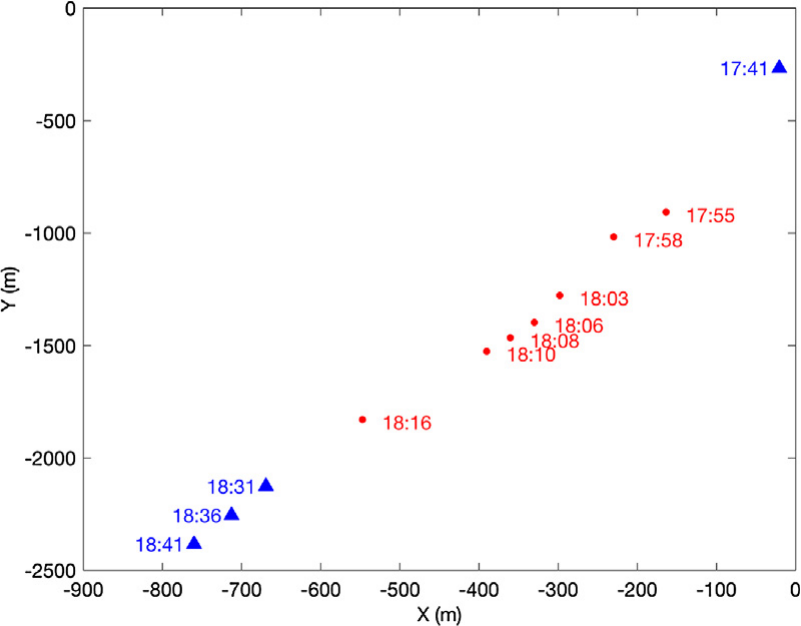
Iceberg positions recorded by an EXITE beacon (blue triangles) reveal a 50 min gap in coverage. During this time, CTD profiles were obtained around the drifting iceberg (red dots). The gap in the EXITE data makes it impossible to estimate the relative distance between the iceberg and CTD measurement location.

Fig. 3 shows iceberg positions (blue triangles) and the locations of CTD measurements made from the small boat (red dots). The distance of each CTD profile to the iceberg could only be characterized qualitatively as near or far. While the small boat was equipped with a functioning radar we lacked the capabilities to use either a software solution or manual data logging of radar-based distances to the iceberg as everyone onboard was engaged in oceanographic data collection and/or vessel operations. Thus, the need for real-time verification of tracker functionality and relatively high frequency position measurements became apparent.

### Design requirements

We set out to develop an iceberg tracking beacon that satisfies the following list of prioritized ‘need-to-have’ requirements. Tracker development was constrained by 1) price; 2) real-time data transmission capabilities over distances on the order of 100 m; 3) 12 h battery life (minimum); 4) time and effort; 5) GPS receiver. Requirement 1 results from budget constraints and the lack of dedicated funding for this project. Requirement 2 stems from the unpredictable nature of icebergs, which can capsize or disintegrate without warning. Requirement 3 takes into consideration the duration of small boat sampling operations in fjords and coastal waters of Greenland, which typically last 8–12 h per day. Requirement 4 reflects the time required to research and test individual components when building custom instruments. As a result, we sought an off-the-shelf solution that required little to no modification for use as an iceberg tracker. GPS receivers are widespread and can be found in many devices but requirement 5 is included for the sake of completeness. A ‘nice-to-have’ requirement was the ability to measure and transmit orientation (pitch, roll, and heading). Orientation data may provide some advance warning of iceberg rolling, which could enhance the safety of small boat operations. Additionally, iceberg rolling was listed as an important reason for ending towing operations [25] and orientation data could increase situational awareness during iceberg management operations.

### Open source Ardupilot autopilots

The growing popularity of drones in recent years has increased the availability of robust and low-cost hardware and software. While the connection may not be immediately apparent, we argue that drone autopilots (also called flight controllers) are naturally suited for high-resolution, real-time iceberg position measurements. To be clear, we did not use a drone to deploy an iceberg tracker [26] nor do we suggest that a drone land on an iceberg. While this could be done, icebergs, especially the smaller ones, can be irregularly shaped and it may not be possible to find an adequate landing area. Furthermore, the additional components needed to complete the drone system (motors and propellers, electric speed controllers, flight battery, and radio control unit - see [27]) would significantly increase the cost of the system. Drone flights would complicate logistics in general and the laws governing drone flights may make some areas off-limits to researchers. Instead of a complete aerial drone system, we used only the autopilot (essentially “brain” of a drone) to view and record real-time observations of iceberg position, speed, and orientation.

Drones, or unmanned aerial vehicles/systems, come in many different configurations and sizes [28,29] yet they all use an autopilot to stabilize the aircraft during flight [30]. Autopilot-stabilized flight distinguishes drones from remote-controlled aircraft, which are piloted entirely by a human operator. There are many autopilots on the market with different capabilities, but those designed for outdoor autonomous flight share the same basic characteristics [27]. Autopilots typically use data from an inertial motion unit (IMU), GPS/GNSS, and a barometer to estimate the ‘state’, or orientation and motion, of the aircraft [31]. Micro-electrical-mechanical systems (MEMS) IMUs are most common and are composed of accelerometers, magnetometers, and gyros. Autopilots also use wireless communications systems to transmit important data, like battery voltage, altitude, position, speed, pitch, roll, and heading, to a ground control station (GCS) running mission planning software.

One of the largest open source drone software solutions is Ardupilot (http://ardupilot.org/). Ardupilot software runs on compatible autopilot boards (hardware) and also includes mission planning software for all major operating systems. Ground control, or mission planning, software includes Mission Planner for Windows (http://ardupilot.org/planner/), APM Planner for Mac OS X (http://ardupilot.org/planner2/), and QGroundControl (QGC) for Windows, Mac OS X, and mobile devices. Mission planning software is used for initial setup of the autopilot board and for viewing real-time telemetered data.

Open-source Ardupilot-compatible autopilot boards include the Pixhawk family of autopilots [32] and several others (see http://ardupilot.org/copter/docs/common-autopilots.html), including the ArduPilot Mega (APM; [30]). We selected the APM for evaluation in this study. The APM has been used by scientists in a variety of vehicles, including fixed-wing aircraft (e.g., [39]), rotary-wing aircraft (e.g., [33]), and autonomous surface vehicles (ASV; e.g., [34]). To the best of our knowledge, this is the first published use of an APM that is used to track a drifting object, like an iceberg.

Here, we show that an APM 2.6 autopilot meets the ‘need-to-have’ and ‘nice-to-have’ requirements for use a high-frequency and real-time iceberg tracker. We purchased an APM 2.6 kit on Amazon for $90, which satisfied Requirement 1. The kit included the APM 2.6 autopilot board and enclosure, external GPS/Compass module (ublox M8N), 915 MHz telemetry radios (ground and rover), power module, and mounting hardware (GPS/Compass mast and vibration dampening plate, which were not used).

The 915 MHz telemetry radios are usually used to transmit data from a flying drone to a fixed GCS. Data packets are sent using the micro aerial vehicle link (MAVlink) protocol [32] and can be viewed in real-time on the GCS. Data are stored in the APM’s onboard memory and as a telemetry log on the ground station computer. The Ardupilot online documentation (http://ardupilot.org/copter/docs/common-sik-telemetry-radio.html) lists the communication range as 300 m, which satisfies Requirement 2. Note that this maximum range assumes an unobstructed line of sight between the antennae and range can be limited or interrupted in the presence of an obstacle. For flying drones, obstacles are typically buildings and trees but for our use case icebergs may limit the effective range. For iceberg tracking, the iceberg itself, as well as other icebergs in the vicinity, may limit communications between the APM and the ground station.

**Fig. 4:**
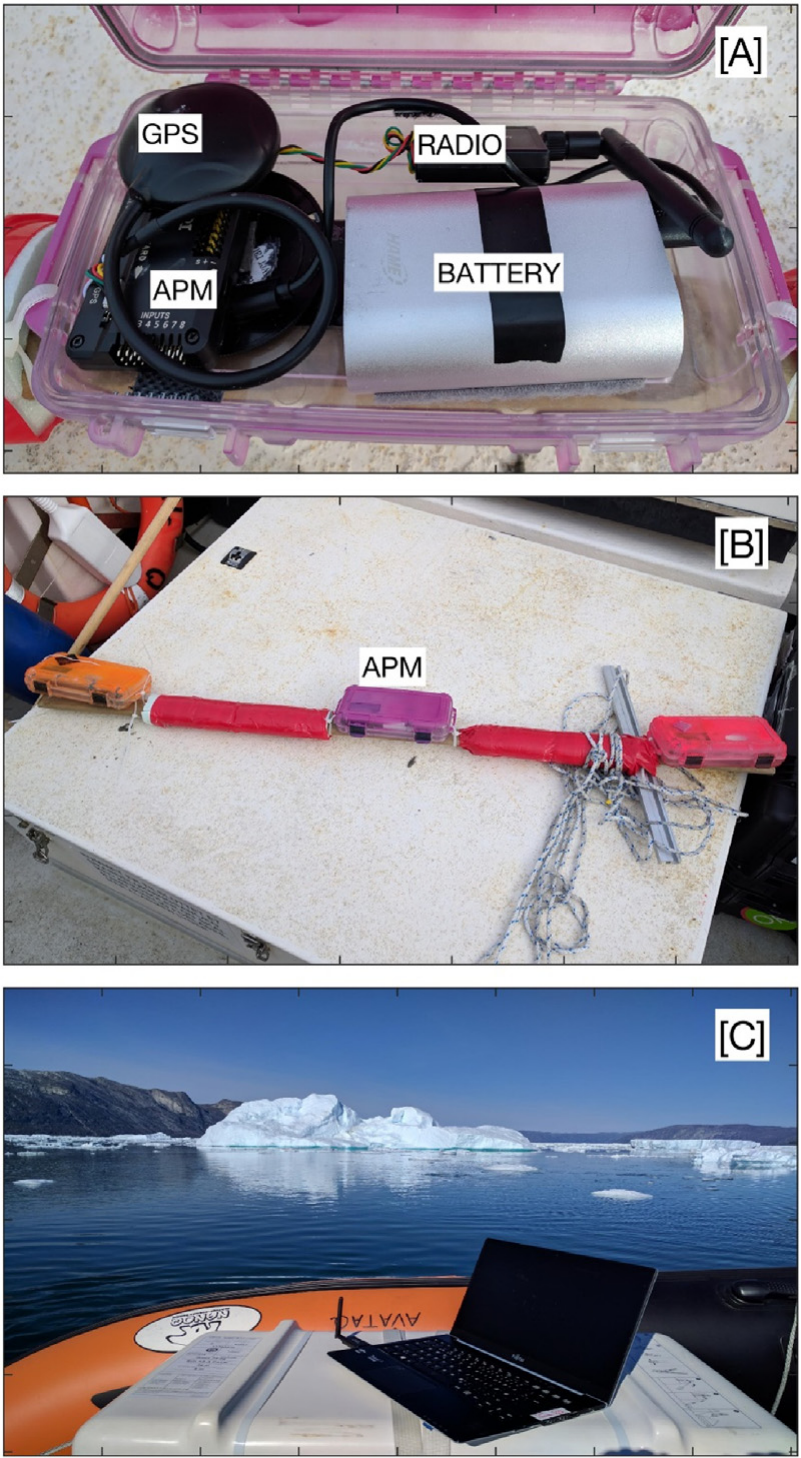
[A] The APM, 915 MHz telemetry radio, 5 V/2 A USB power bank, and external GPS/Compass module installed in a waterproof box. [B] The waterproof box containing the APM is attached to a wooden slat with crossbar. Lengths of rope are tied to either end of the slat to aid in recovery. [C] The ground station computer on the boat receives the telemetry data from the APM on the iceberg seen in the distance.

Autopilots are usually powered from the drone’s flight battery but we powered the APM using an 11 Ah lithium ion (Li-ion) smartphone/tablet powerbank that outputs 5V/2A using a micro USB cable (Fig. 4A). While not extensive, our bench tests satisfied Requirement 3 as this battery consistently powered the APM for at least 12 h, thereby satisfying Requirement 3. The battery indicator reached 50% capacity after approximately 12 h, which suggests a maximum battery life of 24 h.

The GPS/compass module and 915 MHz telemetry radio are plug-and-play components with their appropriate connections labeled on the APM enclosure. We used QGC and the online Ardupilot documentation (http://ardupilot.org/copter/docs/initial-setup.html) to configure the APM and to calibrate its onboard sensors. The plug-and-play components and the online instructions make the APM (and similar autopilots) very easy to configure and use, thereby satisfying Requirement 4. Requirement 5 was satisfied as the APM kit includes a GPS antenna. In addition to the ‘need-to-have’ requirements, the APM also satisfies the ‘nice-to-have’ requirement listed in the previous section. The APM, like all other autopilots, also estimates its orientation (pitch, roll, and heading) and its altitude and transmits these data to the ground station computer.

The APM line of autopilots was officially discontinued but aftermarket ‘clones’ are currently available for sale. Furthermore, we stress that the methodology presented here applies to any Ardupilot-compatible autopilot, and not only the APM 2.6. While Ardupilot compatible autopilots run the same software there are some hardware differences that are worth noting. The onboard storage of the APM was not sufficient to store the relatively large log files (10–25 mb) that are generated during deployments longer than 25 min. The Pixhawk line of autopilots uses a microSD card that greatly increases onboard storage capabilities. While we make every effort to retrieve the tracker we treat it as expendable and rely on the data that are telemetered to the ground station.

### Autopilot configuration for iceberg tracking

The APM, external GPS/compass module, 915 MHz telemetry radio, and 11 Ah Li-ion battery were secured in a small waterproof box using velcro and double-sided tape (Fig. 4A). The waterproof box and its contents were attached to a 1.2 m wooden slat with a 30 cm aluminum cross bar to prevent the assembly from rolling (Fig. 4B). Pipe insulation was taped to the wooden slat to provide enough buoyancy to permit retrieval in the event of an unsuccessful deployment (Fig. 4B). Ropes were tied to either end of the wooden slat to aid in retrieval (Fig. 4B). The total cost of all components was $130 and, therefore, could be treated as disposable if the iceberg capsized or if conditions prohibited a safe retrieval. A standard laptop computer was used as a ground station and was placed on the stern of the vessel at a height of approximately 2 m above the waterline (Fig. 4C).

### Tips and tricks

Normally the data that are telemetered from the APM will not be stored in a log file until the motors on the vehicle have been armed. However, these data can be stored by changing settings in QGC. The following steps are summarized on the Ardusub website (https://www.ardusub.com/operators-manual/logging.html). Power on the APM and connect QGC. In the ‘General’ tab in QGC check the ‘Prompt to save Flight Data log even if vehicle was not armed’ box. The telemetry data will now be saved on the ground control computer in ‘.tlog’ format in the ‘Telemetry’ directory in the ‘Save Path’ in the ‘Vehicle Setup’ tab in QGC. Binary telemetry logs (.tlog) can be viewed in QGC, Mission Planner, and APM Planner and the .tlog binary data can be exported to .mat, .csv, or .kml. As not all of the data stored in the telemetry log are useful for iceberg tracking a custom Matlab script was developed to read relevant data from a .csv file. The script is included in the supplementary material.

### Method validation

**Fig. 5:**
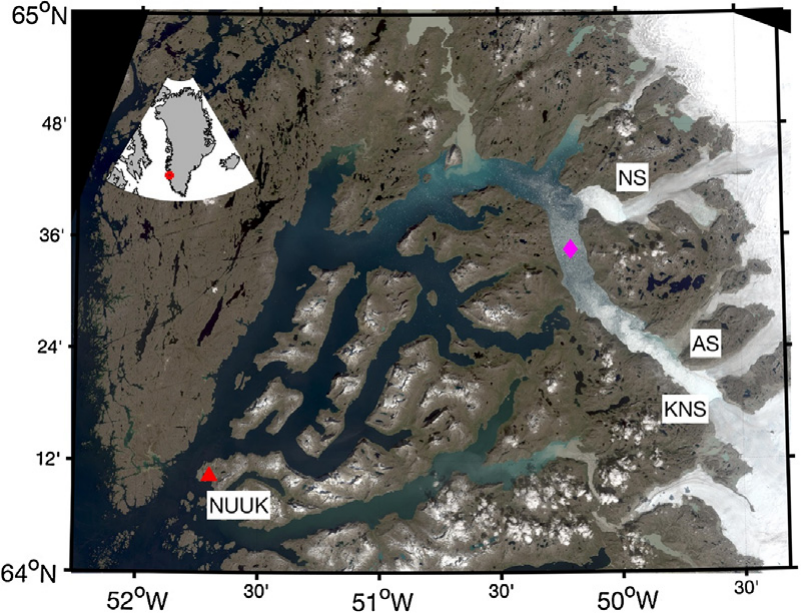
A contrast-enhanced RGB Landsat 8 image of Godthåbsfjord (GF) that was obtained 22 July 2017 (imagery available at http://earthexplorer.usgs.gov/). The red triangle and pink diamond correspond to the city of Nuuk and the iceberg deployment location, respectively. The outlet glaciers of Narssap Sermia (NS), Kangiata Nunata Sermia (KNS), and Akugdlerssup Sermia (AS) are also shown. The inset (upper left) shows the location of GF in Greenland.

**Fig. 6:**
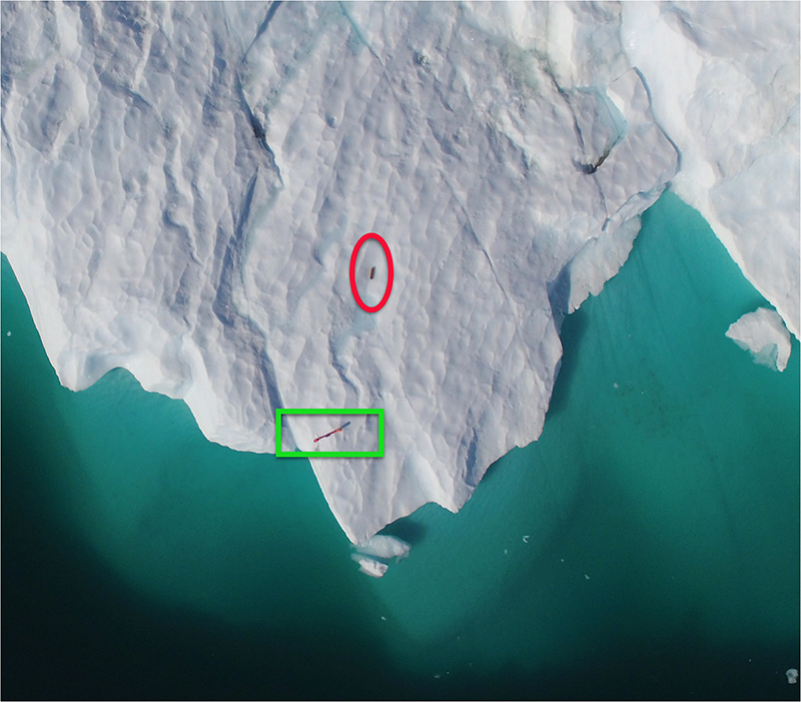
An aerial drone photo of the APM assembly green rectangle) and EXITE beacon (red oval) that were deployed from a small boat onto an 80 m-long iceberg. The APM is attached to a wooden slat that is 1.2 m in length.

The APM’s iceberg tracking capabilities were evaluated in Godthåbsfjord (GF) on 28 August 2017. As no direct funding was available for tracker development these tests were conducted opportunistically during previously planned field sampling. The APM was powered on in the morning before the boat left the harbor and was powered off at the end of the day, approximately 10 h later. Air temperatures varied from 5 to 8 °C on the day of sampling. The APM assembly (Fig. 4B) was deployed on an iceberg and telemetered data to a laptop on a small boat (Fig. 4C). The iceberg was approximately 80 m long and was located in the inner part of GF, approximately 10 km from two tidewater glaciers (Fig. 5). The APM was deployed on the iceberg by maneuvering a small boat (8 m) next to an iceberg long enough to allow a researcher to carefully slide the assembly onto the ice. An EXITE beacon was also deployed by hand on the same iceberg (Fig. 6). The APM collected data on the iceberg for approximately 70 min. The APM was retrieved by snagging one of the ropes with a boat hook and pulling it back onto the boat. The APM assembly remained fixed to the same spot on the iceberg as the aluminum cross bar melted into the ice and given the warm air temperatures it did not refreeze.

The temporal resolution of the data stored in the telemetry log varied from 1 Hz–5 Hz. The telemetry log file was exported to .csv using QGC. The telemetry log file, converted .csv file, and Matlab script used to read the time, GPS position and orientation (pitch, roll, and heading) are included in the Supplementary Material. Note that the telemetry log (.tlog) file can only be viewed in QGC, Mission Planner, or APM Planner, all of which are freely available at http://www.ardupilot.org. GPS coordinates were converted to universal transverse Mercator (UTM) referenced to the world geodetic system 1984 (WGS84) using the ‘deg2utm’ function in Matlab (http://mathworks.com/matlabcentral/fileexchange/10915-deg2utm). Ten second averages of position and orientation data were computed.

The iceberg’s trajectory, as recorded by the APM, during the 70 min test is shown in Fig. 6. A gap of approximately 15 min occurred when line-of-sight communication between the 915 MHz transceivers was interrupted. This interruption could have been prevented by increasing the elevation of the ground station transceiver.

**Fig. 7:**
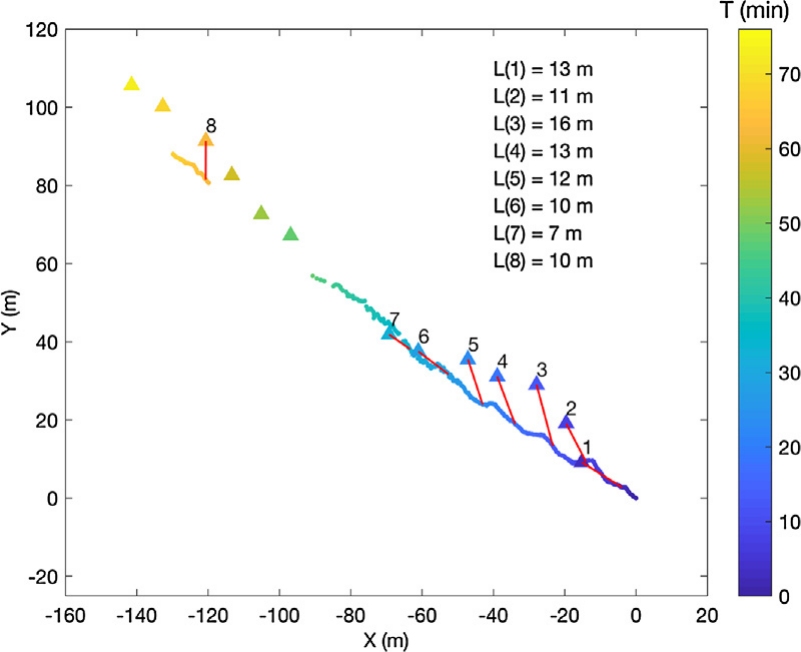
Ten second iceberg positions observed by the APM are indicated by small dots. Positions recorded by an EXITE beacon on the same iceberg are indicated by triangles. All markers are color-coded according to time (in minutes) since APM deployment. EXITE positions with corresponding APM positions are numbered and a red line connects concurrent measurements. The distance between the EXITE and APM for each of these 8 measurements is indicated in the upper right.

APM and EXITE positions are plotted in Fig. 7. Both devices show a gradual drift of the iceberg to the northwest during the 70 min test. The relatively low temporal resolution of the EXITE measurements is apparent when compared to the 10 s APM positions. A 15 min gap occurred in the EXITE record approximately 40 min into the test. The distance between the APM and the EXITE also ranged from 7 to 16 m throughout the test. The APM assembly remained fixed to the ice until retrieval and the EXITE beacon did not appear to move. The differences in position, therefore, can be attributed to multi-path reflection from the iceberg sail or to the position accuracy of the EXITE GPS receiver, which was estimated by Poje et al. [35] as 6.4 m. We did not test the accuracy of the ublox M8N GPS receiver that was supplied with the APM but given that it can used for real-time kinematic GPS applications [36] we assume it is better than that of the Spot Trace used in the EXITE.

**Fig. 8:**
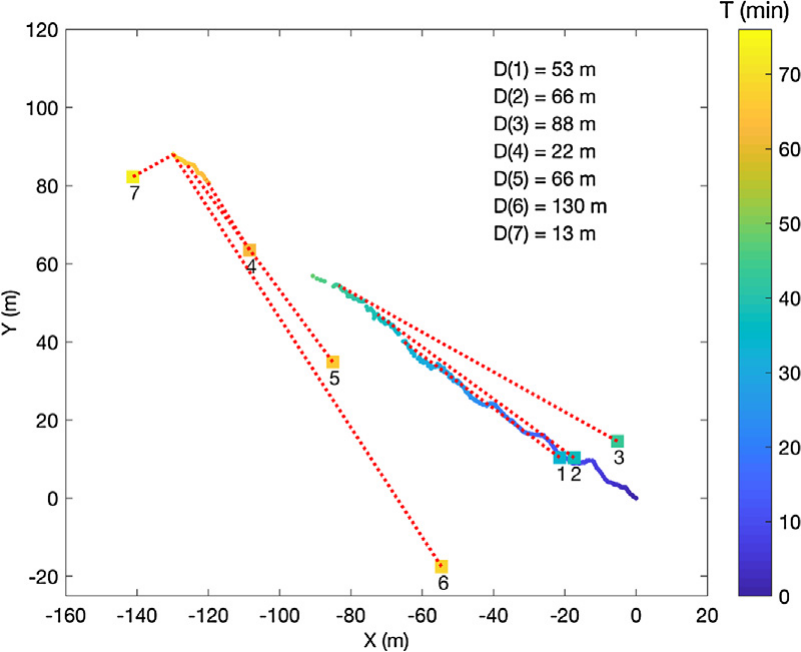
Ten second iceberg positions observed by the APM are indicated by small dots. Locations of CastAway CTD profiles are indicated by the numbered squares. All markers are color-coded according to time (in minutes) since APM deployment. Red dashed lines connect the CTD profile location to the iceberg position at the time of each measurement. The distance, rounded to the nearest meter, of each CTD profile to the APM on the iceberg is displayed in the upper right corner.

After the APM and EXITE were deployed on the iceberg, the small boat slowly drifted away from the iceberg while researchers performed seven CTD profiles. The CastAway has an embedded GPS that stores the location and time of each profile. Fig. 8 shows the CastAway locations and the distances from each profile to the APM on the iceberg for each measurement. CTD profiles were suspended when the data transmission link from the APM to the base station computer was lost and resumed once the connection was reestablished. The GPS time measurement was common to both devices and the distance between each device was estimated by finding the smallest time difference between them. The distance between each GPS location was then computed. These distances ranged from 13 m to 130 m.

**Fig. 9:**
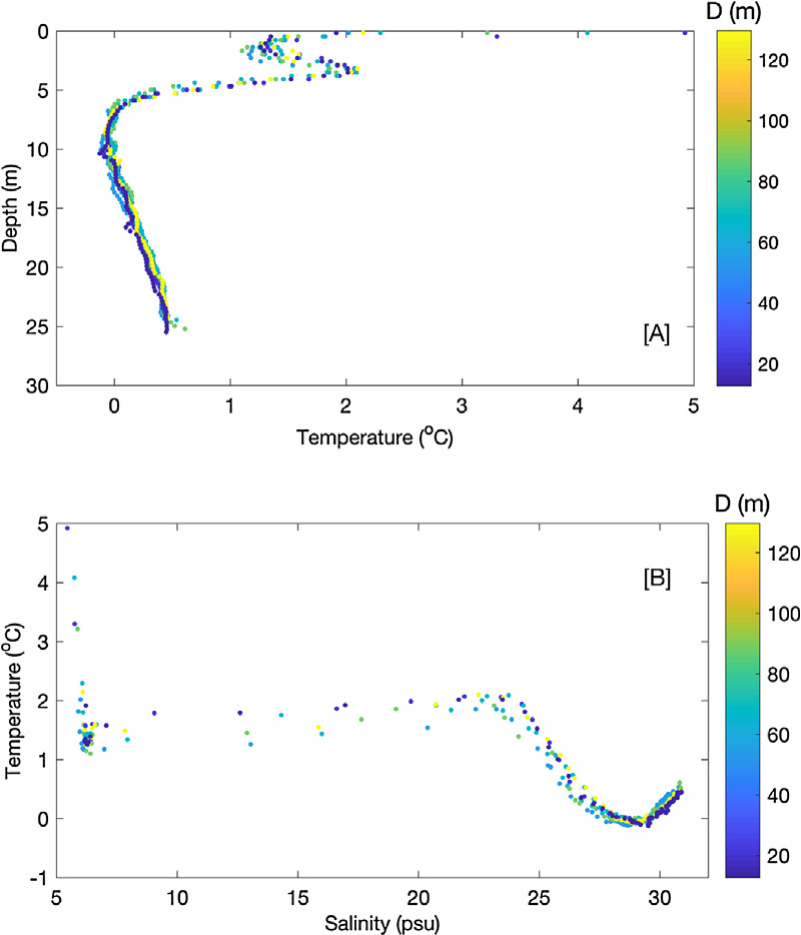
Depth profiles of temperature (A) and temperature/salinity plots (B) from the seven CTD casts that were performed around the drifting iceberg. Measurements are color-coded according to distance from the APM on the iceberg.

A CastAway CTD was used to measure water temperature and salinity from near-surface down to approximately 25 m. The water depth at this location was approximately 600 m. Note that deeper profiles would normally be used to study the meltwater plume of an iceberg of this size but we wanted to collect profiles at multiple locations during the test of the APM. While these CTD data were largely collected to test the APM, Fig. 9 shows depth profiles of temperature and a temperature/salinity (TS) plot that are composed of the seven profiles. The temperature profiles exhibit similar behavior and one of the most notable results is that the warmest measurement also occurred very close to the iceberg (within 13 m; Fig. 9A). However, the APM trajectory in Fig. 8 shows that this CTD profile was performed in front of the iceberg, which highlights the importance of knowing the iceberg’s direction of travel as well as the distance of the observation to the iceberg. The TS plot shows two water masses the summer surface water and the subglacial water [37]. Given the proximity of these measurements to two marine-terminating glaciers and the abundance of ice in the area we do not expect to see clear evidence of iceberg meltwater plumes. An iceberg’s meltwater plume will likely be more recognizable in the outer fjord or on the continental shelf where the overall freshwater content is generally lower [37]. However, we have sufficiently demonstrated the APM’s utility in interpreting oceanographic measurements in the context of a drifting iceberg.

**Fig. 10:**
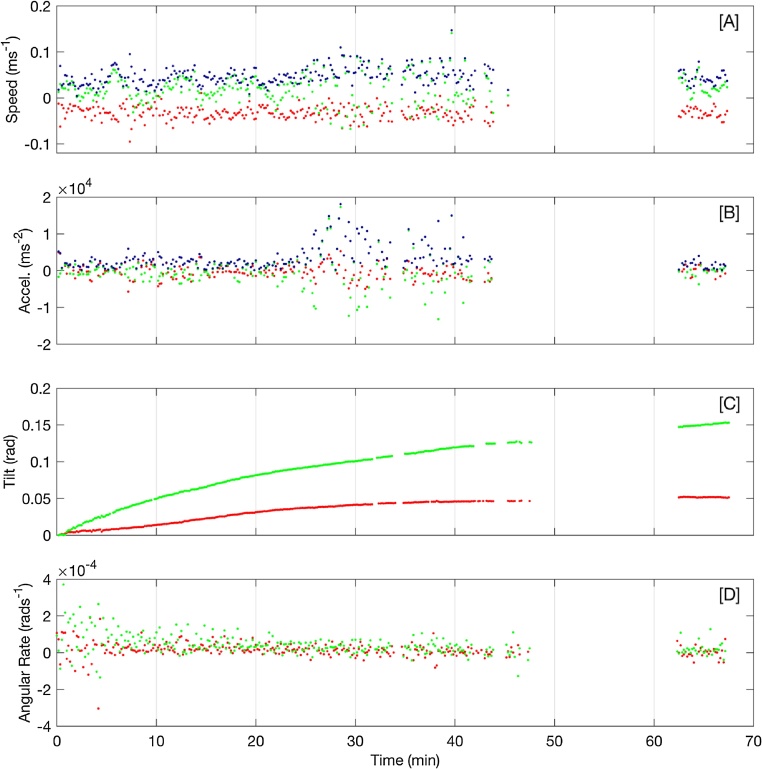
[A–B] Linear speed and acceleration of the iceberg computed from GPS positions. The meridional (north-south), zonal (east-west), and total speed are indicated by red, green, and blue dots, respectively. [C] The iceberg’s pitch and roll are indicated by red and green dots, respectively. [D] Angular acceleration rates computed from the pitch and roll.

Fig. 10 shows iceberg velocity, acceleration, pitch/roll, and angular acceleration rates. Linear velocity and accelerations were computed from UTM positions using central difference schemes. Magnetic heading data were discarded because of magnetic anomalies that could not be removed using standard calibration procedures. As a result, only pitch and roll are presented. Fig. 10A shows that the iceberg’s speed ranged from 0–0.1 m/s throughout the test deployment. The iceberg accelerated approximately 25 min into the test (Fig. 10B). The pitch and roll measurements are referenced to the autopilot’s own orientation and do not translate directly into rotation about a given iceberg axis. However, they show a gradual tilt (Fig. 10C) that was confirmed visually by a change in the iceberg’s waterline. The submerged ice shelf visible in Fig. 6 could have caused significant damage to the small boat if the iceberg rolled during retrieval of the APM and the real-time orientation data provided an extra layer of security.

### Concluding remarks

We showed that the APM, an open-source drone autopilot, can be easily adapted for use as a real-time iceberg tracker. We demonstrated its ability to provide reliable estimates of the distance from an oceanographic measurement to a drifting iceberg and to aid in the interpretation of oceanographic data. The real-time data transmission capabilities of the APM may also prove useful for near-field iceberg management operations. The methods demonstrated here apply to all Ardupilot-compatible open and closed-source autopilots. The battery used here provided over 10 h of continuous operation and larger batteries or 5 V power banks with embedded solar panels could extend measurements to several days. QGroundControl, the open source ground control software used here, can track multiple autopilots simultaneously, which could allow real-time tracking of multiple icebergs.

There are many commercial iceberg trackers with different capabilities and we simply present another tool that fills a specific niche. The APM, however, may also benefit similar research efforts and may also be of use in near-field iceberg management operations (e.g., [6]) and in the navigation of unmanned vehicles around icebergs (e.g., [38]), especially vehicles using the MAVlink communications protocol. Given its low cost and ease of use, its utility can be assessed by researchers and industry professionals without a serious investment in time or money. Furthermore, the system presented here is based on open source hardware and software and is supported by excellent online documentation and user forums. While we use the system ‘as-is’, the open source framework can allow other users to ‘hack’ it to fit their specific needs.

http://asp-net.org.

## References

[1] Ardhuin F., Tournadre J., Queffeulou P., Girard-Ardhuin F., Collard F. (2011). Observation and parameterization of small icebergs: drifting breakwaters in the southern ocean. Ocean Model..

[2] Helly J.J., Kaufmann R.S., Stephenson G.R., Vernet M. (2012). Cooling, dilution and mixing of ocean water by free-drifting icebergs in the Weddell Sea. Deep Sea Res. II.

[3] Smith K.L., Sherman A.D., Shaw T.J., Sprintall J. (2013). Icebergs as unique Lagrangian ecosystems in Polar seas. Ann. Rev. Mar. Sci..

[4] Gagnon R.E., Wang J. (2012). Numerical simulations of a tanker collision with a bergy bit incorporating hydrodynamics, a validated ice model and damage to the vessel. Cold Reg. Sci. Technol..

[5] Yulmetov R., Marchenko A., Løset S. (2016). Iceberg and sea ice drift tracking and analysis off north-east Greenland. Ocean. Eng..

[6] Holub C., Matskevitch D., Kokkinis T., Shafrova S. (2018). Near-field ice management tactics - simulation and field testing. Cold Reg. Sci. Technol..

[7] Bigg G. (2016). Icebergs: Their Science and Links to Global Change.

[8] Helly J.J., Kaufmann R.S., Vernet M., Stephenson G.R. (2011). Spatial characterization of the meltwater field from icebergs in the Weddell Sea. Proc. Natl. Acad. Sci. U. S. A..

[9] Yankovsky A.E., Yashayaev I. (2014). Surface buoyant plumes from melting icebergs in the Labrador Sea. Deep-Sea Res. I.

[10] FitzMaurice A.C., Cenedese C., Straneo F. (2017). Nonlinear response of iceberg side melting to ocean currents. Geophys. Res. Lett..

[11] Chan H.C., Davies T.W., Hall P. (1997). Iceberg tracking using HF surface wave radar. MTS/IEEE Conference Proceedings.

[12] Dowdeswell J.A., Whittington R.J., Hodkins R. (1992). The sizes, frequencies, and freeboards of East Greenland icebergs using ship radar and sextant. J. Geophys. Res..

[13] Kjerstad Ø.K., Løset S., Skjetne R., Skarbø R.A. (2018). An ice-drift estimation algorithm using ship radar and ship motion measurements. IEEE Trans. Geosci. Remote. Sens..

[14] Turnbull I.A., Fournier N., Stolwijk M., Fosnaes T., McGonigal D. (2015). Operational iceberg drift forecasting in Northwest Greenland. Cold Reg. Sci. Technol..

[15] Voytenko D., Dixon T.H., Luther M.E., Lembke C., Howat I.M., de la Pena S. (2015). Observations of inertial currents in a lagoon in southeastern Iceland using terrestrial radar interferometry and automated iceberg tracking. Comput. Geosci..

[16] Carlson D.F., Boone W., Meire L., Abermann J., Rysgaard S. (2017). Bergy bit and melt water trajectories in Godthåbsfjord (SW Greenland) observed by the expendable ice tracker. Front. Mar. Sci..

[17] Crawford A.J., Wadhams P., Wagner T.J.W., Stern A., Abrahamsen E.P. (2016). Journey of an Arctic ice island. Oceanography.

[18] Jones D.H., Gudmundsson G.H. (2015). Tracking B-31 iceberg with two aircraft-deployed sensors. Nat. Hazards Earth Syst. Sci..

[19] Larsen P.-H., Overgaard Hansen M., Buus-Hinkler J., Harnvig Krane K., Sønderskov C. (2015). Field tracking (GPS) of ten icebergs in eastern Baffin Bay, offshore Upernavik, Northwest Greenland. J. Glaciol..

[20] Sulak D.J., Sutherland D.A., Enderlin E.M., Stearns L.A., Hamilton G.S. (2017). Iceberg properties and distributions in three Greenlandic fjords using satellite imagery. Ann. Glaciol..

[21] Sutherland D.A., Roth G.E., Hamilton G.S., Mernild S.H., Stearns L.A., Straneo F. (2014). Quantifying flow regimes in a Greenland glacial fjord using iceberg drifters. Geophys. Res. Lett..

[22] Gladstone R., Bigg G.R. (2002). Satellite tracking of icebergs in the Weddell Sea. Antarct. Sci..

[23] Eik K., Gudmestad O.T. (2010). Iceberg management and impact on design of offshore structures. Cold Reg. Sci. Technol..

[24] Lund B., Graber H.C., Persson P.O.G., Smith M., Doble M., Thomson J., Wadhams P. (2018). Arctic sea ice drift measured by shipboard marine radar. J. Geophys. Res..

[25] Sudom D., Timco G., Tivy A. (2014). Iceberg sightings, shapes, and management techniques for offshore Newfoundland and Labrador: historical data and future applications. 2014 Oceans- St. Johns.

[26] McGill P.R., Reisenbichler K.R., Etchemendy S.A., Dawe T.C., Hobson B.W. (2011). Aerial surveys and tagging of free-drifting icebergs using an unmanned aerial vehicle (UAV). Deep-Sea Res.-II..

[27] Liu P., Chen A.Y., Huang Y.-N., Han J.-Y., Lai J.-S., Kang S.-C., Wu T.-H., Wen M.-C., Tsai M.-H. (2014). A review of rotorcraft unmanned aerial vehicle developments and applications in civil engineering. Smart Struct. Syst..

[28] Colomina I., Molina P. (2014). Unmanned aerial systems for photogrammetry and remote sensing: a review. ISPRS J. Photogramm. Remote Sens..

[29] Klemas V. (2015). Coastal and environmental remote sensing from unmanned aerial vehicles: an overview. J. Coast. Res..

[30] Ebeid E., Skriver M., Terkildsen K.H., Jensen K., Schultz U.P. (2018). A survey of open-source UAV flight controllers and flight simulators. Microprocess. Microsyst..

[31] Kortunov V.I., Mazurenko O.V., Gorbenko A.V., Mohammed W. (2015). Review and comparative analysis of mini- and micro-UAV autopilots. Proceedings of the IEEE 3^rd^ International Conference Actual Problems of Unmanned Aerial Vehicles Developments (APUAVD).

[32] Meier L., Tanskanen P., Heng L., Hee Lee G., Fraundorfer F., Pollefeys M. (2012). PIXHAWK: a micro aerial vehicle design for autonomous flight using onboard computer vision. Auton. Robots.

[33] Ventura D., Bruno M., Lasinio G.J., Belluscio A., Ardizzone G. (2016). A low-cost drone based application for identifying and mapping of coastal fish nursery grounds. Estuarine Coast. Shelf Sci..

[34] Kimball P., Bailey J., Das S., Geyer R., Harrison T., Kunz C., Manganini K., Mankoff K., Samuelson K., Sayre-McCord T., Straneo F., Traykovski P., Singh H. (2014). The WHOI jetyack: an autono mous surface vehicle for oceanographic research in shallow or dangerous waters. 2014 IEEE/OES Autonomous Underwater Vehicles (AUV).

[35] Poje A.C., Ozgokmen T.M., Lipphardt B.L., Haus B.K., Ryan E.H., Haza A.C., Jacobs G.A., Reniers A.J.H.M., Olascoaga M.J., Novelli G., Griffa A., Beron-Vera F.J., Chen S.S., Coelho E., Hogan P.J., Kirwan Jr A.D., Huntley H.S., Mariano A.J. (2014). Submesoscale dispersion in the vicinity of the *Deepwater Horizon* spill. Proc. Natl. Acad. Sci. U. S. A..

[36] Skoglund M., Petig T., Vedder B., Eriksson H., Schiller E.M. (2016). Static and dynamic performance evaluation of low-cost RTK GPS receivers. 2016 IEEE Intelligent Vehicles Symposium (IV).

[37] Mortensen J., Lennert K., Bendtsen J., Rysgaard S. (2010). Heat sources for glacial melt in a sub-Arctic fjord (Godthåbsfjord) in contact with the Greenland Ice Sheet. J. Geophys. Res..

[38] Kimball P.W., Rock S.M. (2015). Mapping of translating, rotating icebergs with an autonomous underwater vehicle. IEEE J. Ocean. Eng..

[39] Ryan J.C., Hubbard A.L., Box J.E., Todd J., Christoffersen P., Carr J.R., Holt T.O., Snooke N. (2018). UAV photogrammetry and structure from motion to assess calving dynamics at Store Glacier, a large outlet draining the Greenland ice sheet. Cryosphere.

