# Supplementary material for: Adapting open-source drone autopilots for real-time iceberg observations
Source: MethodsX. 2018 Sep 6;5:1059–72. doi: 10.1016/j.mex.2018.09.003 (PMC6139390; doi:10.1016/j.mex.2018.09.003)
Supplement: Supplementary file 1 [file mmc1.docx]

**Supplementary material *and/or* Additional information:**

The iceberg tracking data used to validate the method and to make Figure 3 are provided in the telemetry log file – 2017-08-28 15-16-58.tlog. This file can be viewed in Mission Planner, QGroundControl, and APM Planner. These data were converted to .csv format in Mission Planner. The Matlab script apm_tel_log_reader.m reads the .csv file into Matlab variables.
